# Systematic review of strategies to increase use of oral rehydration solution at the household level

**DOI:** 10.1186/1471-2458-13-S3-S28

**Published:** 2013-09-17

**Authors:** Lindsey M Lenters, Jai K Das, Zulfiqar A Bhutta

**Affiliations:** 1 Centre for Global Child Health, The Hospital for Sick Children, Toronto, ON, Canada; 2 Division of Woman and Child Health, Aga Khan University, Karachi, Sindh, Pakistan

## Abstract

**Background:**

Diarrhea is one of the major causes of death in children under five years of age, disproportionately affecting children in low- and middle-income countries. Treatment of diarrhea with oral rehydration solution addresses dehydration and reduces diarrhea related deaths. The World Health Organization Programme for the Control of Diarrhoeal Disease began in 1978 and while global ORS access rates have improved substantially over the past forty years, rates of ORS use have stagnated. Investigation is required to understand which interventions are effective in promoting the use of ORS, and where there are gaps in the literature.

**Methods:**

We conducted a systematic search of peer-reviewed and grey literature and included interventions to promote the use of ORS for the treatment of acute diarrhea in children under 6 years. We used a standardized grading format based on the Child Health Epidemiology Research Group guidelines and performed meta-analysis for all categories with more than one data point.

**Results:**

We identified 19 studies for abstraction. For co-promotion of zinc and ORS, mothers in the intervention group were 1.82 (95% CI 1.17, 2.85) times more likely to use ORS to treat their child’s diarrhea episode than mothers in the comparison group. Meta-analysis of ORS social marketing and mass media strategies indicates that mothers exposed to messages were 2.05 (95% CI, 0.78, 5.42) times more likely to use ORS to treat their child’s diarrhea episode than unexposed mothers. However, this is not statistically significant. Both meta-analysis had significant heterogeneity and were graded as moderate/low and low quality, respectively.

**Conclusions:**

We found few studies of interventions to promote the use of ORS; many categories of interventions had only one study. While there are some promising results, this analysis reinforces the need for further investigation into approaches to increasing ORS use.

## Background

Diarrhea is one of the major causes of death in children under five years of age, leading to an estimated 1.071 million annual deaths and disproportionately affecting children in low- and middle-income countries [1]. In patients with diarrhea, the cause of death is almost always due to fluid loss and dehydration [2]. This can be addressed through fluid therapy in the form of oral rehydration solution - a simple, cost-effective treatment that was proven to be effective during a cholera epidemic in Bangladesh in the 1970s [3]. Treatment of diarrhea with oral rehydration solution (ORS) can remedy 90% of dehydration from diarrhea. ORS is the cornerstone of diarrhea treatment, according to the World Health Organization (WHO), whereas antibiotic treatment in addition to ORS is only indicated in cases of cholera or bloody diarrhea [2].

The WHO Programme for the Control of Diarrhoeal Disease began in 1978. Activities in diarrheal disease control programs vary widely from country to country and have included social marketing and mass media campaigns, the involvement of political figures and religious leaders, educational campaigns in schools, training of partly skilled health care workers, changes to medical school curricula, distribution schemes, as well as the establishment of outpatient oral rehydration centers [2].

A 2010 systematic review by Munos et al. indicated that universal coverage with ORS would reduce diarrhea related deaths by 93% [4]. While ORS access rates have improved substantially over the past forty years, use rates of ORS have stagnated. Access to ORS in developing countries increased drastically in the 1980s, from an estimated 5% in 1982 to 61% in 1988 for children under 5 years of age [5]. Over the same time period, the percentage of children with diarrhea in the last two weeks receiving ORS or recommended home fluids during the diarrhea episode increased from approximately 0% in 1982 to 32% in 1988 [5]. However, global ORS use rates have not changed substantially since the late-1980s, remaining at about 30% [6,7].

In a recent paper, Boschi-Pinto et al. found that more than half of the countries included in their analysis had no significant improvement, or had a reduction in the coverage of oral rehydration therapy for diarrhea (17/29 countries) [8], where coverage is defined as the proportion of a population in need of an intervention who receive the intervention. An analysis conducted by Ram et al., which included a broader definition of ORT (oral rehydration solution, recommend home solution or increased fluids) demonstrated similar results [6]. The reasons for this plateau are complex, and may be in part due to declining funding for diarrhea control programs [7]. Other contributing factors include lack of political commitment and insufficient resources and infrastructure, or socio-cultural factors such as the lack of perceived benefit of ORS, given that ORS does not decrease the volume of stool output during the diarrhea episode [9]. There are forces at play at the household and community level that can be addressed through community-level programming to promote the use of ORS; and a synthesis of evidence around these promotion approaches is needed.

**Figure 1:**
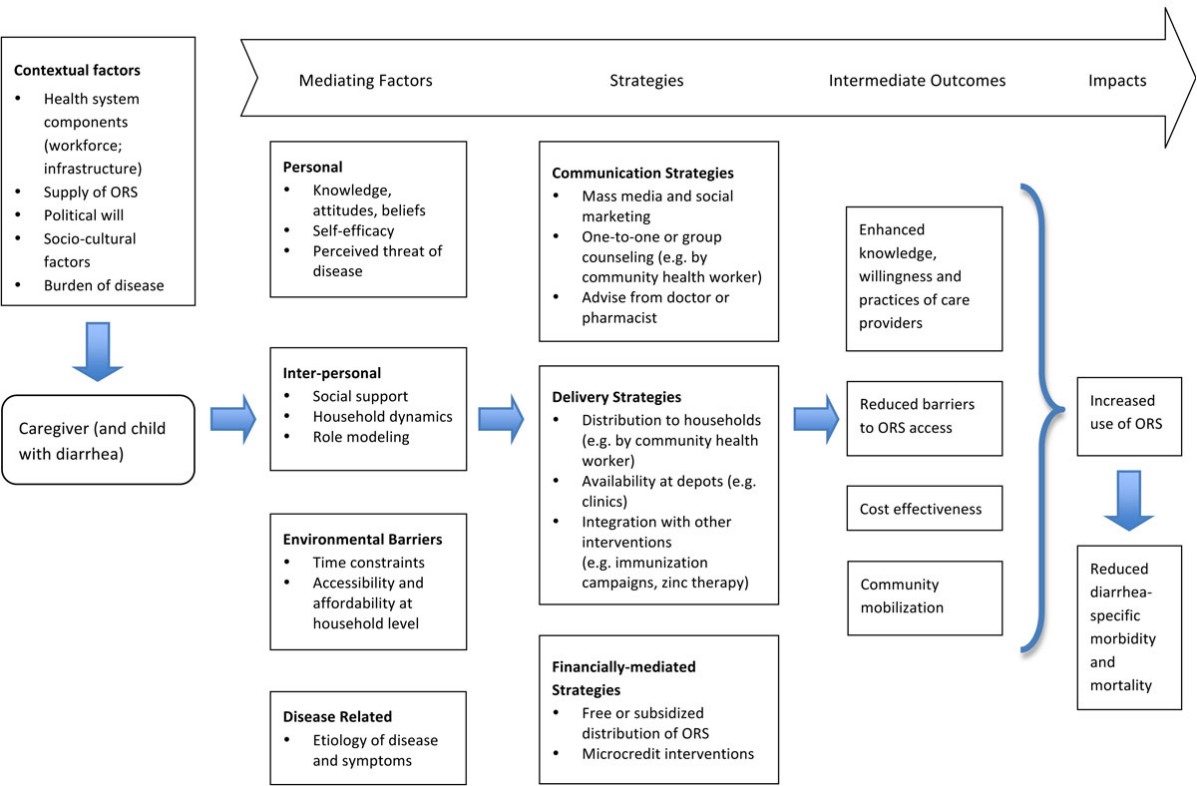
**Conceptual model: strategies to promote the use of oral rehydration solution at the household level** ORS: Oral rehydration solution

This review aims to evaluate the effectiveness of strategies to promote and scale-up ORS for the treatment of acute childhood diarrhea. Collating the available evidence will also shed light on areas where more research is needed. Based on the various strategies identified through the literature search, a conceptual framework has been developed to help elucidate the processes by which ORS interventions influence caregivers’ knowledge and behaviours, within a particular environmental context, ultimately impacting use rates of ORS and reducing rates of diarrhea-related mortality (see figure 1).

## Methods

### Searching

We conducted a systematic literature search of peer-reviewed and grey literature, which included community-based interventions to promote the use of ORS for the treatment of acute diarrhea in children under 6 years of age. Studies were identified through searches of Medline, WHO Regional Databases, Cochrane libraries, OpenGrey, and Grey Literature Report. Additional studies were identified through hand searches of key references lists and Google scholar®. Searches were initially conducted on March 15, 2012 and updated on July 18, 2012. Studies in English were included, and the literature search covered studies published from 1970 to July 2012. The search strategy included combinations of the terms: fluid therapy, oral rehydration solution, diarrhea, community health workers, community health education, mass media, social marketing, health promotion, zinc therapeutic use. The Medline search strategy is included in additional file 1.

### Inclusion/exclusion criteria

We included randomized controlled trials, quasi-experimental and observational studies looking at the effectiveness of interventions aimed at promoting the use of oral rehydration solution for the treatment of mild or moderate acute diarrhea in children under 6 years of age. Included studies could either be an intervention focused solely on ORS, or could be part of a broader strategy. We limited our search to studies conducted in low- and middle-income countries. We did not define the categories of interventions in advance, but chose broad search terms that would capture a range of programs and interventions. Only studies using WHO-defined ORS were considered eligible for this review (because of the timeframe of studies included, this covers both standard and low-osmolarity ORS). We did not include studies looking at the use of home-prepared sugar-salt solutions or home-available fluids. The outcome of interest was ORS use: whether the care provider had used ORS to treat their child’s current or most recent episode of diarrhea.

### Data extraction and validity assessment

Studies were screened using a two-stage process: one investigator screened titles and abstracts in order to select studies published in English that potentially met the inclusion criteria. Subsequently, two investigators reviewed the shortlist of studies to assess whether studies met the inclusion criteria and contained the relevant outcome indicator. Disagreements between investigators were resolved through discussion. Studies were abstracted separately by two reviewers into a standardized excel spreadsheet.

The Child Health Epidemiology Research Group (CHERG) adaptation of the GRADE criteria [10] was applied to assess the quality of individual studies, and the overall category-level data. Studies were classified as high, moderate, low or very low quality. Randomized controlled trials were initially graded as high quality, quasi-experimental studies as moderate quality, and observational studies as low quality. The quality rating of each study was increased or decreased as indicated by CHERG guidelines [10] through an assessment of study methods, sample size, risk of bias, and generalizability.

Quality assessment was also performed at the outcome level, considering the overall quality of the body of evidence along the dimensions of: volume and consistency of results across studies, the size of the pooled effect estimate, and the strength of evidence for the effect estimate as indicated by the p-value. The quality of the body of evidence was graded as high, moderate, low or very low.

### Analysis

Studies graded as very low quality were excluded from the meta-analysis, as they were not deemed to be sufficiently reliable. For each category of intervention with more than one study, a meta-analysis was conducted using Review Manager version 5.1®. Due to the range in quality of evidence found through this review, we performed a separate meta-analysis on randomized-controlled trials and quasi-experimental, as well as a meta-analysis on observational studies. We applied the generic inverse variance method to all meta-analyses and report the random effects pooled relative risk (DerSimonian Laird method) and 95% confidence interval. We made an a priori decision to apply a random effects model to all meta-analyses, as we were not expecting the effects being estimated in each study to be identical.

## Results

### Search results

**Figure 2:**
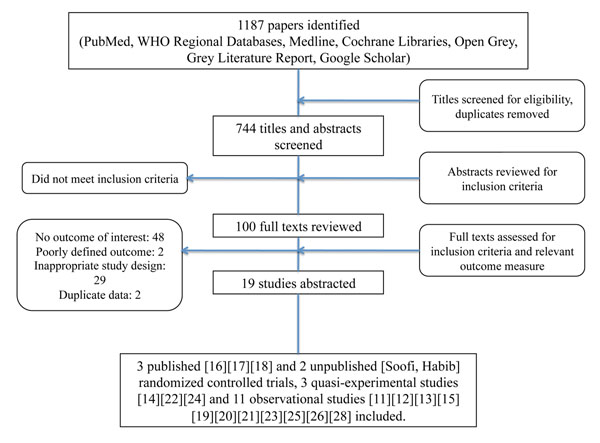
Flow diagram showing identification of studies included in the review

Electronic and hand searches returned 1187 studies. After removing duplicates and excluding studies based on reviewing titles and abstracts for relevant inclusion criteria, 100 full text articles were reviewed (figure 2). From the full text articles reviewed 19 met the review inclusion criteria and had the relevant outcome measure (refer to additional file 2). A range of types of interventions were found and were organized into the following categories: co-promotion of zinc and ORS, co-packaging of zinc and ORS, social marketing and mass media, distribution strategies, community-based education, microcredit interventions and multi-pronged nationwide strategies. After excluding studies graded as very low quality [11-13], we were able to conduct meta-analyses on two categories of ORS strategies: co-promotion of zinc and ORS as well as social marketing and mass media. The characteristics of the studies meeting the inclusion criteria for this review can be found in additional file 3.

Meta-analysis for other intervention categories was not possible. After excluding very low quality studies and after separating the observational studies from the quasi-experimental and randomized controlled trials, we were left with only one data point in each category. Additionally, there are two low quality studies, a community education intervention [14] and a complex National Control of Diarrheal Disease intervention [15] that could not be pooled because the studies did not provide sufficient detail to be able to calculate the relative risk.

The results of the meta-analyses are presented below. Characteristics and outcome data for the individual studies included in the meta-analysis, as well as the studies meeting the inclusion criteria which were not included in any meta-analysis, can be found in additional file 3.

### Zinc therapy for diarrhea

We included four randomized controlled trials evaluating the effectiveness of promoting zinc therapy along with ORS for the treatment of diarrhea in a community setting. Three are published studies [16-18] and one is a recent study that has not yet been published, included with permission from the author (Soofi, S.). We also included an unpublished study investigating the effect of co-packaging dispersible zinc tablets with ORS and promoting the product through social marketing and mass media channels (Habib, A.). This study meets the inclusion criteria, yet was not included in the meta-analysis. The intervention was considered to be substantially different from the other zinc interventions, given that the zinc and ORS were marketed as part of a single product, the “diarrhea pack”.

**Figure 3:**
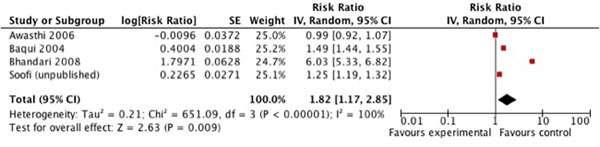
Forest plot for the effect of co-promotion of zinc and ORS on ORS use

Meta-analysis of the studies investigating the co-promotion of zinc and ORS indicate that mothers in the intervention arm were 1.82 (95% CI 1.17, 2.85) times more likely to treat their child’s diarrhea episode than mothers in the comparison group (figure 3). The quality of evidence for this intervention type was graded as moderate/low quality (table 1).

**Table 1:** Quality assessment of evidence at the category level

	Quality Assessment					Summary of Findings			
				**Directness**		**No of events**			

**No of studies**	**Design**	**Limitations**	**Consistency**	**Generalizability to population of interest**	**Generalizability to intervention of interest**	**Intervention**	**Control**	**Relative Risk**	**95% Confidence Interval**

**Strategy: Co-promotion of Zinc and ORS (Moderate/low outcome specific quality)**									

4	RCT/cRCT	None	3 of 4 studies showed beneficial effect; heterogeneity in meta-analysis	No major limitations	No major limitations	5345	3895	1.82	[1.17, 2.85]

**Strategy: Social marketing and mass media (Low outcome specific quality)**									

3	Observational	Variation in study design and quality	All show beneficial effect; heterogeneity in meta-analysis; not statistically significant	No major limitations	No major limitations	1530	804	2.05	[0.78, 5.42]

cRCT: cluster-randomized controlled trial

ORS: Oral rehydration solution

### Social marketing and mass media

**Figure 4:**
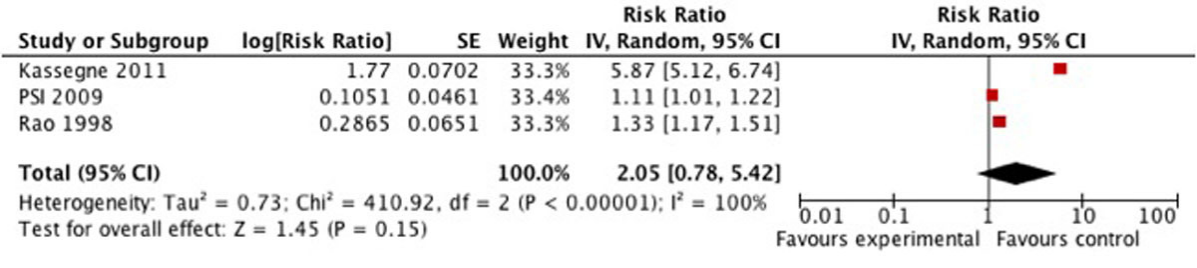
Forest plot for the effect of social marketing and mass media strategies on ORS use

We found three observational studies looking at the impact of social marketing and mass media strategies on mother’s use of ORS to treat their child’s diarrhea episode [19-21]. The meta-analysis indicates that mothers who were exposed to media and social marketing strategies through radio, television and cinema spots as well as community outreach activities and print materials were 2.05 (95% CI 0.78, 5.42) times more likely to use ORS to treat their child’s diarrhea episode than mothers who were not exposed, or had low levels of exposure (figure 4). This evidence is not statistically significant (p=0.15) and the outcome level data was graded as low quality (table 1).

## Discussion

Overall we found very few trials or high quality observational studies investigating the effectiveness of interventions to promote the use of ORS. The paucity of high quality research on interventions to improve ORS use limits the insight that can be gleaned from this analysis, nevertheless the results of this review highlight promising areas as well as gaps that need to be addressed through further research.

The strongest evidence found through this systematic review relates to the co-promotion of zinc and ORS for the treatment of childhood diarrhea, largely because this evidence is derived from four randomized controlled trials. However, the generalizability of this analysis is limited by the fact that there was a geographical bias towards South Asia. Additionally, the effect appears to be driven by one of the four studies. There is also evidence suggesting beneficial effects of social marketing and mass media for promoting the use of ORS to treat diarrhea, yet the meta-analysis results are not statistically significant. Challenges with measuring exposure to mass media may have influenced the results.

The remaining studies included in this review indicate beneficial effects to varying degrees of interventions such as community education [14,22], distribution strategies [23,24], microcredit interventions [25] and complex strategies such as a National Control of Diarrheal Disease program [26] or a national ORS program coupled with other public health and education interventions [15]. However, we were unable to conduct meta-analyses on these strategies as explained above.

There are several limitations in this review. One issue with this analysis, inherent in the design of the individual studies, is the inability to separate out the effects of multiple interventions occurring simultaneously. In some cases the intervention is designed around a multi-pronged approach, for example, the combined delivery of zinc and ORS. Furthermore, other health interventions occurring in the community could impact on the mother’s use of ORS to treat their child’s diarrhea episode. This is particularly problematic if researchers are not aware of, or are not measuring these other interventions. The majority of studies in this review did not provide a robust description of services offered at baseline in the intervention or comparison group, and did not explore this potential confounding variable in their analyses.

An additional issue relates to the approach to the meta-analysis. Studies included in this review were categorized under a primary intervention, yet most included additional intervention activities. As much as possible, we tried to group studies according to similar profiles of interventions; however, there is likely to be some heterogeneity in terms of the types of intervention. The impact of the intervention may be due not just the main intervention, but to other activities as well. Full details of the intervention activities are available in additional file 3.

There is an expanse of descriptive data available on the topic of patterns ORS use; yet very few rigorous investigations of interventions to scale up the use of ORS. We only found randomized controlled trials on zinc interventions. The remaining body of literature is quasi-experimental or observational, and the study designs were largely before/after study designs or program/non-program comparison designs. A major limitation of before/after studies is that without a control group, it is not possible to determine whether the observed change in the outcome is due to the program, or to other factors [27]. Studies using a comparison of program and non-program areas increase the plausibility of causal effect; however, there are many issues with this study design. For example, it is increasingly uncommon to find comparison sites that have no other health programs underway making it difficult to have truly ‘untouched’ comparison group [27].

While observational studies can be rigorously executed, not all of the studies meeting the inclusion criteria for this review applied techniques that would increase the quality, for example adjusting for potential confounding factors. However, in the meta-analysis of social marketing and mass media campaigns, two of the three studies adjusted for major sociodemographic characteristics [19,20]; the adjusted effect estimate for the third study [21] was not provided in a usable format, however the adjusted and unadjusted effect estimates presented in the paper were similar, thus we felt confident in using the unadjusted data.

As this review relied on published and grey literature, it may not accurately reflect the landscape of country-level experiences with ORS promotion. The review collated data only from studies measuring the effect of a specific intervention on the use of ORS, meaning that where studies of this nature do not exist, a country will not be represented. However, there is an upcoming country-level case study analysis of the promotion of ORS use (Saul Morris, personal communication August 2012) that will be able to fill in some of the gaps not be covered by this review.

ORS is clearly efficacious in preventing diarrhea-related mortality, yet there are barriers towards promoting its use, which have led to stagnated global rates of ORS use for the treatment of childhood diarrhea. High quality research is needed to understand how best to promote the uptake of ORS for the treatment of acute childhood diarrhea, not only at the household consumption level, but from multiple vantage points within the system. Moreover, in the studies included in this review the baseline population level coverage of ORS was not systematically reported. This is a key piece of information to report, as different interventions may be more effective at raising use from low to moderate levels, while others may be more successful at raising use from moderate to high levels. Finally, more research is needed in regions other than South Asia, such as Sub-Saharan Africa, given that the effect of behaviour change interventions will likely differ across settings.

Beyond researching the promotion of ORS use at the household level, research is needed to explore other aspects of the equation necessary for scaling up ORS use, including strategies to educate health care providers about ORS treatment, methods to ensure a reliable supply of ORS at no cost or low-cost through the public sector, or methods to make the sale of ORS profitable through financing mechanisms that are attractive to private sector investment [2].

## Conclusions

Most child deaths occur due to a small number of conditions that are preventable, even in the poorest settings, through interventions that are well known, affordable and deliverable via simple technologies [8]. Oral rehydration solution is one such example, yet there are significant barriers that have contributed to stagnated rates of ORS use globally.

The systematic review returned studies looking at a variety of interventions to increase the use of ORS to treat diarrhea in children. Strategies included zinc supplementation for the treatment of diarrhea, social marketing and mass media, community education, microcredit interventions, distribution programs and multi-pronged nationwide strategies. A multi-pronged approach, including elements of mass media, health force training and novel products such as zinc may have the potential to increase the use of ORS to treat diarrhea episodes in children. While the interventions in this review show promise, firm conclusions cannot be drawn due to issues with the small volume of the evidence and high levels of heterogeneity within the meta-analyses. Research is needed specifically investigating strategies to scale-up the use of ORS, looking at the system from multiple vantage points, in a range of settings where ORS use has been historically low.

## Authors' contributions

LML was involved in the literature search, data abstraction, data analysis and writing the manuscript. JKD was involved in the review conceptualization, data abstraction, data analysis and writing the manuscript. ZAB made significant contributions to the conceptualization of this review as well as manuscript revisions.

## Competing interests

The authors declare they have no conflict of interest.

## Supplementary Material

Additional file 1 Medline Search Strategy Click here for file

Additional file 2 Data abstraction table and list of excluded studies Click here for file

Additional file 3 Table of characteristics of the studies included in the review Click here for file

## Declarations

The publication costs for this supplement were funded by a grant from the Bill & Melinda Gates Foundation to the US Fund for UNICEF (grant 43386 to "Promote evidence-based decision making in designing maternal, neonatal, and child health interventions in low- and middle-income countries”). The Supplement Editor is the principal investigator and lead in the development of the Lives Saved Tool (LiST), supported by grant 43386. He declares that he has no competing interests.

This article has been published as part of *BMC Public Health* Volume 13 Supplement 3, 2013: The Lives Saved Tool in 2013: new capabilities and applications. The full contents of the supplement are available online at http://www.biomedcentral.com/bmcpublichealth/supplements/13/S3.
