# Supplementary material for: Systematic review of strategies to increase use of oral rehydration solution at the household level
Source: BMC Public Health. 2013 Sep 17;13(Suppl 3):S28. doi: 10.1186/1471-2458-13-S3-S28 (PMC3847633; doi:10.1186/1471-2458-13-S3-S28)
Supplement: Additional file 1 — Medline Search Strategy [file 1471-2458-13-S3-S28-S1.docx]

**Medline Search Strategy**

1 exp Fluid Therapy/ (13169)

2 Rehydration Solutions/ (1137)

3 (fluid adj4 therap*).mp. (15390)

4 (ORS or ORT).ti,ab. (9842)

5 (oral adj4 rehydrat*).mp. (2354)

6 (alhydrate or dioralyte or elotrans or gastrolyte or "ges 45" or glucosolan or infalyte or lytren or pedialyte or rehydralyte or "rehydration solution*" or rehydron or reidrax or resol or ricelyte).mp. (1875)

7 or/1-6 (25615)

8 Diarrhea/ (36134)

9 (diarrhoea or diarrhea).mp. (77837)

10 8 or 9 (77837)

11 7 and 10 (3056)

12 (infan* or newborn* or new-born* or neonat* or baby* or babies or child* or kid or kids or toddler* or boy* or girl* or p?ediatric*).mp. (2335654)

13 11 and 12 (2402)

14 Diarrhea, Infantile/ (6360)

15 7 and 14 (1002)

16 13 or 15 (2402)

17 Community Health Workers/ (2797)

18 community health services/ or child health services/ or community networks/ (45758)

19 "delivery of health care"/ or health services accessibility/ or healthcare disparities/ (103658)

20 (community or communities).ti,ab. (264317)

21 Health Promotion/ (46469)

22 health education/ or patient education as topic/ (113292)

23 exp mass media/ or pamphlets/ (39878)

24 Social Marketing/ (1840)

25 ((health or wellness) adj2 (promotion* or campaign* or educat*)).mp. (118775)

26 (social adj2 (market* or medi*)).mp. (15211)

27 (television* or TV* or radio* or movie* or pamphlet*).mp. (1042187)

28 ((patient* or famil??? or parent* or mother*) adj2 (educat* or informat*)).mp. (100137)

29 Zinc/tu, th [Therapeutic Use, Therapy] (2314)

30 zinc.ti,ab. (65952)

31 or/17-30 (1683329)

32 16 and 31 (614)
