# Supplementary material for: Systematic review of strategies to increase use of oral rehydration solution at the household level
Source: BMC Public Health. 2013 Sep 17;13(Suppl 3):S28. doi: 10.1186/1471-2458-13-S3-S28 (PMC3847633; doi:10.1186/1471-2458-13-S3-S28)
Supplement: Additional file 3 — Table of characteristics of the studies included in the review [file 1471-2458-13-S3-S28-S3.docx]

| **Study** | **Description** | **Participants** | **Intervention** | **Risk Ratio  (95% CI)** | **Quality Assessment** |
| --- | --- | --- | --- | --- | --- |
| **Co-promotion of Zinc and ORS** | | | | | |
| **Awasthi 2006** | Block randomized trial in Brazil, Ethiopia, Egypt, India and the Philippines in community settings to assess the impact of zinc supplementation and promotion on use of ORS, antibiotics or anti-diarrheals in children with acute watery diarrhea. | Children 2 to 59 months with diarrhea | ORS and zinc given (20 mg orally, once daily for 14 days) along with counseling and site-specific, culturally appropriate messages for zinc usage | 2.55 (2.47, 2.63) | High |
| **Bhandari 2008** | Cluster randomized controlled trial in rural India from 2005 to 2006 to evaluate if education and provision of zinc supplements to caregivers is effective in the treatment of acute diarrhea and if there are adverse effects on the use of oral rehydration salts. | Children 1 to 59 months | ORS and 20 mg zinc for 14 days (10mg 1-5 mo) available from CHW, public, and private health centers | 1.49 (1.44, 1.55) | Moderate |
| **Baqui 2004** | A cluster randomized controlled trial in Bangladesh to evaluate the effectiveness of zinc supplementation and the impact on ORS use in a community setting. | Children 3 to 59 months | 20 mg Zn for 14 days available from CHWs in intervention in addition to ORS and advise on appropriate feeding practices | 1.25 (1.19, 1.32) | High |
| **Soofi (unpublished)** | A cluster randomized controlled trial in Pakistan from 2006 to 2007 to evaluate the effectiveness of zinc supplementation for 14 days and to explore changes in use of ORS in the community. | Children 2 to 59 months with acute diarrhea | Zinc tablets and ORS provided through public health care facilities, lady health workers, private practitioners, pharmacies and drug stores. Health care providers given education from the zinc promotion team. Large-scale zinc promotion campaign also undertaken. | 0.99 (0.92, 1.07) | Moderate /high |
|  |  |  |  |  |  |
| **Larson 2011** | An ecological study in four representative populations in Bangladesh from 2006 to 2009 to monitor the national Scaling Up of Zinc for Young Children project | Children 2 to 59 months with diarrhea | Mass media campaign; private sector partnership for dispersible tablet formulation, brand name and packaging design, pricing, over-the-counter waiver, and a permit to advertise nationally on TV and radio | 1.07 (1.03, 1.11) | Low |
| **Co-packaging and Co-promotion of Zinc and ORS** | | | | | |
| **Habib (unpublished)** | A cluster randomized controlled trial in Pakistan to evaluate the effectiveness of zinc supplementation for 14 days and the impact on ORS use in a community setting. | Children under 5 years of age | 'Diarrhea pack' distributed through CHWs and pharmacies; meetings with community leaders, health care providers and community members; print material distributed to health care providers; counseling at household level by CHWs. | 6.03 (5.33, 6.82) | Moderate |
| **Social Marketing and Mass Media** | | | | | |
| **Kassegne 2011** | Two cross-sectional surveys conducted in Burundi between 2004 and 2007 to evaluate a social marketing intervention to promote ORS | Children under 5 years old | Radio spots and community outreach activities in schools and health centers; health workers, vendors, and pharmacy employees trained in the promotion and use of ORS; educational and promotional materials distributed. | 5.87 (5.12, 6.74) | Moderate |
| **Rao 1998** | An analysis of data from India's 1992-3 National Family Health Survey to explore the impact of exposure to mass media and social marketing activities on mother's treatment of diarrhea in children with ORS | Children under 5 years old | Radio and television spots; longer documentaries shown in cinema halls; community-level activities in rural areas including film shows, drama and song performances, exhibitions, group meetings, and training camps. | 1.33 (1.17, 1.51) | Moderate |
| **PSI 2009** | Two cross-sectional surveys conducted in Malawi in 2005 and 2008 to evaluate the impact of a social marketing and media campaign on mothers' use of ORS during their child's diarrhea episode | Children under 5 years old | Radio adverts to inform about ORS, radio spots and jingle; targeted interpersonal activities | 1.11 (1.02, 1.22) | Low |
| **Community Education** | | | | | |
| **Pahwa 2010** | Quasi-experimental study of a nine-month community-based health and nutrition-education intervention in an urban slum in India. | Children age >12 to 71 months | Education on the necessity of oral rehydration through group discussions at community centers and small groups within the urban jhuggis/slums. | 3.34 (2.36, 4.74) | Low |
| **Jintaganont 1988** | Quasi-experimental study in rural Thailand over a 7-month intervention period in 1986 to assess the impact of an oral rehydration therapy program. | Children under 5 years old | Village health communicators and volunteers trained on diarrhea management and promotion of ORS; monthly meetings with groups of women to promote the use of ORS; religious leaders mobilized | Unable to calculate RR | Low |
| **Distribution Strategies** | | | | | |
| **Rahaman 1979** | A longitudinal cohort study conducted in rural Bangladesh from 1985 to 1986 to assess the effect of a community distribution and promotion of ORS program | Children 1- 4 years old | Free provision of ORS at community distribution points in intervention site; 18 'depot-holders' trained to educate and supply ORS to community; ORS promoted in public venues; information given to students and teachers in primary schools. | 2.08 (1.77, 2.44) | Low |
| **Kumar 1987** | A quasi-experimental study conducted in rural India over two years to evaluate the role of ORS in reducing diarrhea-related deaths in children in the community | Children below 6 years | WHO-ORS available from primary health care workers or literate volunteers | 4.27 (4.02, 4.54) | Low |
| **Complex Interventions** | | | | | |
| **Langsten 1995** | A population based survey conducted between 1979 and 1991 in Egypt showing trends in treatment of diarrheal disease following the implementation of the National Control of Diarrheal Diseases Project. | Children under 5 years old who had ever had diarrhea | Increased in-country production and distribution of ORS; television spots featuring well-known character; training health workers on large scale establishment of rehydration rooms in hospitals and clinics. | 3.50 (3.24, 3.79) | Low |
| **Gutierrez 1996** | A longitudinal population based survey conducted in Mexico between 1986 and 1993 to assess the impact of a multi-pronged education, sanitation and selected medical care practices intervention on child health outcomes. | Children under 5 years old | ORS packets distributed by the national health system starting in 1984 following a comprehensive social communication program and training strategy at the medical unit level and community level | Unable to calculate RR | Low |
| **Microcredit Interventions** | | | | | |
| **MkNelly 1998** | Two cross-sectional surveys conducted in Ghana in 1993 and 1996 to assess the impact of a conditional microcredit program on various health outcomes including ORS use. | Children 12 - 23 months | Small-scale loans combined with education in the basics of health, nutrition, family planning and small business skills | 5.23 (3.19, 8.56) | Low |
